# It is not NOD2 — genetic and clinical risk factors for postoperative complications following ileocolic resection in Crohn’s disease

**DOI:** 10.1007/s00384-022-04223-6

**Published:** 2022-08-01

**Authors:** Josefine Schardey, Sophie Zehl, Alina S. Kappenberger, Petra Zimmermann, Florian Beigel, Tobias S. Schiergens, Michael S. Kasparek, Florian Kühn, Jens Werner, Ulrich Wirth

**Affiliations:** 1 Department of General, Visceral and Transplant Surgery, Ludwig-Maximilians-University Munich, Marchioninistr. 15, 81377 Munich, Germany; 2 Department of Medicine II, University Hospital, LMU Munich, Munich, Germany; 3 Department of Visceral Surgery, Josephinum, Munich, Germany

**Keywords:** Crohn’s disease, Ileocolic resection, NOD2, Wound healing, Inflammatory bowel disease

## Abstract

**Purpose:**

To evaluate the role of the nucleotide oligomerization domain 2 (NOD2) mutation status and other risk factors for the incidence of postoperative complications after ileocolic resection for Crohn’s disease (CD).

**Methods:**

Data of 138 patients consecutively undergoing ileocolic resection for CD at a tertiary academic referral center were retrospectively analyzed including single nucleotide polymorphism (SNP) data of the NOD2 gene. Uni- and multivariate regression analysis was performed to identify factors associated with increased risk of severe postoperative complications.

**Results:**

From 114 patients (83%), the NOD2 mutation status was available. Of these, 60 (53%) had a NOD2 wildtype, whereas eleven (10%) were homozygous for the high risk p.Leu1007fsX1008 (rs2066847) variant. Major postoperative complications occurred in 28 patients (20%). Twenty-seven of these (96%) were intraabdominal septic complications such as anastomotic leakage or abscess. Male gender (*P* = 0.029; OR 3.052, the duration of CD (time [months] from initial diagnosis of CD to surgery; *P* = 0.001; OR 1.009), previous abdominal surgery for CD (*P* = 0.017; OR 3.49), and the presence of enteric fistulas (*P* = 0.023; OR 3.21) were identified as independent risk factors for major postoperative complications. Homozygosity for the NOD2 high-risk variant p.Leu1007fsX1008 did not show increased postoperative morbidity in the short and long-term outcome.

**Conclusions:**

We could detect independent risk factors for major postoperative complications after ileocolic resection for Crohn’s disease. However, patients with the high-risk variant p.Leu1007fsX1008 of the NOD2 gene did not show increased postoperative morbidity.

## Introduction

Crohn’s disease (CD) is characterized by a chronic relapsing disease course based on a chronic inflammation of the intestine, frequently resulting in a stricturing or penetrating disease phenotype [1]. Despite encouraging improvements in medical treatment of CD by immunomodulators and biologicals [2], surgical therapy is still required in the majority of patients [2–4]. About one-third of all CD patients eventually undergo surgery for CD [5]. The most common procedures represent surgery for perianal fistulas and bowel resections [6, 7]. Compared to other benign disease, in CD, the rate of postoperative morbidity remains high: a recent prospective study reports a postoperative morbidity rate of 36% [8]. Furthermore, not only in short-term but also in long-term outcome patients experiencing postoperative complications are at risk for undergoing repeated abdominal surgery in the future [5].

Thus, it is important to identify high-risk patients in order to reduce morbidity. So far, several clinical, serological, and genetic factors have been investigated regarding prognosis in CD. Specific polymorphisms of the NOD2 gene have been associated with younger CD onset, ileal disease and ileocolectomies, and some with increased recurrence after surgery [9, 10]. Among these, three variants of the NOD2 gene, p.Arg702Trp, p.Gly908Arg, and p.Leu1007fsX1008, have been reported to be associated with a complicated disease course particularly with a stricturing and penetrating disease behavior [1, 11]. In this context, the frameshift mutation p.Leu1007fsX1008 in exon 11 of the NOD2 gene has been successfully used as a marker for therapeutic decisions [1]. In the gastrointestinal mucosa, NOD2 is widely expressed on various cell types [12]. It is part of a family of intracellular pathogen recognition receptors (PRRs) and its signaling influences microbial activity and prevents pathogenic invasion [12].

Whether it might be a useful predictor for postoperative morbidity has not been conclusively elucidated yet. NOD2-deficient mice exhibit impaired healing of the ileocolonic anastomosis with an intestine-specific effect [13]. Therefore, the aim of this study was to investigate the role of the high risk frameshift mutation of NOD2 p.Leu1007fsX1008 in the context of perioperative morbidity in CD patients undergoing ileocolic resections.

## Patients and methods

### Design and study population

Demographic and perioperative data of a previously well-described cohort [1] of patients undergoing ileocolic resection for histologically proven CD between 2001 and 2010 at the Department of Surgery, Ludwig-Maximilians-University of Munich, Germany, were retrospectively analyzed. A follow-up of these patients was performed until 2021. Patients undergoing repeat (neo-)ileocolic resection were excluded from the study. This retrospective study was approved by the Ethics committee of the Ludwig-Maximilians-University of Munich, Germany (723–16). This retrospective cohort study was conducted according to the Strengthening the Reporting of Observational Studies in Epidemiology (STROBE) guidelines [14].

### Data collection

Preoperative assessment included patient demographics (age, gender) and relevant clinical characteristics such as immunemodulatory or immunosuppressive medication and smoking history. Furthermore, for genotype–phenotype analysis, mutations of the NOD2 gene (the three common CD-associated NOD2 mutations (p.Arg702Trp (rs2066844), p.Gly908Arg (rs2066845), and p.Leu1007fsX1008 (rs2066847)) were derived from the local CD database as previously described [1]. Patient’s disease phenotype was classified according to *Montreal classification* [15]. Among perioperative parameters, the technique of surgery (open vs. laparoscopic assisted) and the estimated intraoperative blood loss (EBL) were assessed. The standard reconstruction technique at our institution is a handsewn double-layer side-to-side anastomosis with a running suture in open and laparoscopic assisted ileocolic resection. From the standardized pathology reports, the resection margins were classified as inflammation-free or not. Postoperative complications were assessed according to the validated Clavien-Dindo classification [16]. Major postoperative complications were defined as those greater than Clavien-Dindo II, and this was defined as main outcome measure as at least some kind of invasive re-intervention was required in these patients. Septic intraabdominal complications were classified as described: anastomotic leakage [17], intraabdominal abscess, and intestinal fistula [6]. For follow-up analysis, a number of different immunosuppressant and immunomodulatory therapies as well as further abdominal surgeries were assessed.

### Statistical analysis

Results were expressed as numbers and percentages or median and range (minimum and maximum). In univariate analysis, dependent on the variable’s character, chi-square-test, Fisher’s exact test (low frequency), or Mann-Withney test for non-normally distributed values were applied. Significant variables were entered into the multivariate analysis; for this purpose, stepwise logistic regression analysis was performed. Odds ratios (OR) with their 95% confidence-intervals (95% CI) were calculated. Concerning significance levels, *P* < 0.05 was regarded as statistically significant. For statistical analysis, SPSS software (version 28.0, IBM, Chicago, USA) was used.

## Results

### Study population and perioperative characteristics

Overall, 138 patients were included in the present study (75 women and 63 men). The clinical baseline and perioperative characteristics are shown in Table 1. The median age at the time of surgery was 35 years (15–69), and the median time span from initial diagnosis of CD to surgery was 50 (0–368) months. Disease localization was limited to ileal (L1) and ileocolic (L3) phenotype in 78 (57%) and 60 (44%) patients, respectively. Overall, 86 patients (62%) had immunosuppressive therapy preoperatively (Table 1). Thirty-one patients (22%) had previous abdominal surgery for CD other than ileocolic resection, and 45 patients (33%) had fistulating disease. Among these, entero-enteric and entero-vesical fistulas were the most common (*N* = 33; 73% of all fistulas).

**Table 1:** Clinical characteristics and univariate analysis for severe postoperative complications

	All patients *N* (%) or range	No complications *N* (%) or range	Complications *N* (%) or range	*P*-value
No. of patients	138 (100)	110 (80)	28 (20)	
Median age *	35 (15–69)	33 (15–69)	39 (20–58)	0.346
Age at initial diagnosis of CD*	25 (8–61)	25 (8–61)	25 (8–53)	0.459
Duration of CD (months)*	50 (0–368)	39 (0–291)	99 (2–368)	0.001
*Montreal Classification*				
* Age of onset*				
A1 below 16 years	20 (14.5)	16 (14.5)	4 (14.3)	
A2 between 17 and 40 years	95 (68.8)	75 (68.2)	20 (71.4)	
A3 above 40 years	23 (16.7)	19 (17.3)	4 (14.3)	0.926
*Localization*				
L1 ileal	78 (56.5)	68 (61.8)	10 (35.7)	
L2 colonic	0	0	0	
L3 ileocolonic	60 (43.5)	42 (33.2)	18 (64.3)	0.013
*Disease type*				
B1 non-stricturing/penetrating	1 (0.7)	1 (0.9)	0	
B2 stricturing	70 (51)	59 (54)	11 (39)	
B3 penetrating	67 (49)	50 (45)	17 (61)	0.377
*Gender*				
Female	75 (54.3)	65 (59.1)	10 (35.7)	
Male	63 (45.7)	45 (40.9)	18 (64.3)	0.034
Body mass index (kg/m^2^)*	22 (13–37)	22 (15–36)	23 (13–37)	0.717
Smoker	50 (36.2)	37 (33.6)	13 (46.4)	0.271
*Immunosuppressive therapy*				
Azathioprine	46 (33.3)	42 (38.2)	4 (14.3)	
TNF-antibody	29 (21.0)	21 (19.1)	8 (28.6)	
Systemic steroids	47 (34.1)	29 (26.4)	7 (25.0)	0.097
NOD2 mutation (*N* = 114)^**^	11 (9.6)	8 (9)	3 (12)	0.717
Preoperative hemoglobin (g/dL)	12.9 (5.8–17.1)	12.7 (7.4–17.1)	14.1 (5.8–16.3)	0.015
Preoperative albumin (g/dL)	4.3 (3.5–5.0)	4.3 (3.6–5.0)	4.3 (3.5–4.8)	0.944
Previous abdominal surgery	31 (22.5)	20 (18.2)	11 (39.3)	0.018
Fistulas	45 (32.6)	31 (28.2)	14 (50.0)	0.041
*Type of surgery*				
Emergency surgery	8 (6)	4 (4)	4 (14)	0.053
Open	80 (58.0)	60 (54.5)	20 (71.4)	
Laparoscopic assisted	58 (42.0)	50 (45.5)	8 (38.6)	0.106
Converted	7 (12.1)	7 (14.0)	0	0.577
Estimated blood loss*	200 (20–1200)	200 (20–1000)	200 (20–1200)	0.183
Resection margin with inflammation	30 (21.7)	23 (20.9)	7 (25.0)	0.616

^*^Median and [range]; ^**^homozygous form of the p.Leu1007fsX1008 (rs2066847) variant

From 114 patients (83%), the NOD2 frameshift mutation status was available (Table 2). Of these, 60 (53%) had a NOD2 wildtype, whereas eleven (10%) were homozygous for the p.Leu1007fsX1008 variant and 16 patients (14%) were heterozygous. The SNPs Arg702Trp (rs2066844) and p.Gly908Arg (rs2066845) were less frequent, with only one (1%) homozygous patient each (Table 2), and with 10 (9%) and 7 (6%) heterozygous patients, respectively*.*

**Table 2:** Distribution of NOD2 polymorphisms

NOD2 allele	Number of patients (%)
All patients	114 (100)
Wild-type allele	60 (53)
p.Leu1007fsX1008 heterozygote	16 (14)
p.Leu1007fsX1008 homozygote	11 (10)
p.Arg702Trp heterozygote	10 (9)
p.Gly908Arg heterozygote	7 (6)
p.Gly908Arg and p.Arg702Trp	4 (4)
p.Leu1007fsX1008 and p.Arg702Trp	2 (2)
p.Leu1007fsX1008 and G908R	2 (2)
p.Arg702Trp homozygote	1 (1)
p.Gly908Arg homozygote	1 (1)

Ten patients (7%) were observed to have a preoperative hemoglobin level below 10 g/dL, and two patients (1%) below 8 g/dL. No patient exhibited hypoalbuminemia. In eight patients (6%), ileocolic resection was performed as emergency surgery, and all of these procedures were carried out as open surgery. No patient received a primary ostomy. In 65 patients (47%), the procedure was performed laparoscopically assisted, seven patients of those had to be converted to open surgery. Patients undergoing laparoscopic surgery were characterized by a significantly lower EBL (150 mL vs. 291 mL; *P* = 0.01) and a lower prevalence of intestinal fistulas (9% vs. 59%; *P* = 0.01) as well as lower severe morbidity (12% vs. 27%; *P* = 0.034). The overall median blood loss was 200 mL (20–1200 mL). In 30 patients (22%), the resection margin of the bowel showed signs of CD-associated inflammation, but this was not significantly correlated to ileocolic disease localization (*P* = 1.0) (Table 1).

### Postoperative morbidity

There was no postoperative mortality in our study. Overall, 55 patients (40%) experienced postoperative complications, 28 of these (20% overall) major postoperative morbidity (Clavien Dindo ≥ 3a) (Table 3). Besides one patient with postoperative hemorrhage and minor septic complication, all of these patients had developed severe intrabdominal septic complications. In 22 patients, re-operation was necessary due to these complications mostly due to anastomotic leak, abscess, and fistula formation. Consecutively, two of them suffered of multi-organ failure and had to be referred to an intensive care unit (Table 3). In five patients, surgical re-intervention led to the creation of an ileostomy. In six patients, CT-guided intervention was performed due to intraabdominal abscess (Table 3).

**Table 3:** Type and incidence of postoperative complications according to the Clavien-Dindo classification

Complications (Clavien Dindo grade)	Number of patients (% of complications overall)
Overall	55 (100)
Grade I	12 (23)
Grade II	15 (27)
Grade III	
Grade IIIA	6 (10)
Grade IIIB	20 (36)*
Grade IV	
Grade IVA	0 (0)
Grade IVB	2 (4)*
Grade V	0 (0)

^*^Overall, 22 patients underwent re-operation

### Univariate and multivariate risk factor analysis

The univariate risk factor analysis for severe postoperative complications is presented in Table 1. Duration of CD, male gender, history of previous abdominal surgery, ileocolic localization (L3), preoperative Hb concentrations, and fistulizing disease were factors significantly associated with major morbidity (Mann–Whitney test, Fisher’s exact/χ^2^ all *P* < 0.05), while there was a trend towards an increased risk in patients who underwent emergency surgery (*P* = 0.053).

Immunosuppressive therapy or immunomodulators (steroids, azathioprine, infliximab, adalimumab), smoking, having the homozygous high-risk NOD2 variant p.Leu1007fsX1008 (rs2066847), and resection margins with inflammation did not predict major postoperative morbidity.

In multivariate analysis, only male gender, duration of CD (time span from initial diagnosis of CD to surgery), previous abdominal surgery for CD other than ileocolic resection, and the presence of fistulas were independent risk factors for the incidence of severe postoperative complications (Table 4). This risk was about threefold higher in men (OR 3.1), patients undergone previous surgery (OR 3.49), or with fistulas (OR 3.2). Every year of CD duration increased the odds of morbidity by 11% (0.9% per month).

**Table 4:** Multivariate analysis for severe postoperative complications

	Postoperative severe morbidity (*N* = 28)		
	*P*-value	Odds ratio	95% CI
Male gender	0.028	3.052	1.118–8.331
Duration of CD (months)	0.001	1.009	1.004–1.015
Previous abdominal surgery	0.019	3.222	1.251–9.733
Fistulae	0.023	3.201	1.177–8.703

### Long-term follow- up after surgery

Follow-up was available in *N* = 89/138 cases (64.5%) with a median duration of 123 (12–864) months (Table 5). Of these patients, *N* = 21 needed further surgery (23.6%) and *N* = 55 needed immunosuppressive or immune-modulatory therapy (61.8%). The median amount of immunosuppressive or immune-modulatory therapy was 1.0 (1–5). The need for immunosuppressive or immune-modulatory therapy over time was not different between patients with or without homozygous NOD2 SNP (χ^2^: *P* = 0.554), smoking or non-smoking patients (χ^2^: *P* = 0.952) or patients with or without fistulating disease (χ^2^: *P* = 0.424). Patients with homozygous NOD2 SNP did not need further surgical treatment significantly more frequent compared to other patients (χ^2^: *P* = 0.471) as well as smoker (χ^2^: *P* = 0.313) or patients with fistulating disease (χ^2^: *P* = 0.684).

**Table 5:** Long-term follow-up data

Follow-up rate *n* (%)		89 (64.5%)
Follow-up duration (median, range)		123 (12–864)
Reoperation *n* (%)		21 (23.6%)
Number of different immunosuppressive or immune-modulatory therapies	No therapy	34
	1 therapy	27
	2 therapies	11
	3 therapies	8
	> 3 therapies	9

## Discussion

To our knowledge, this is the first study to investigate the impact of high-risk SNP p.Leu1007fsX1008 (rs2066847) status on postoperative surgical outcome in a CD cohort undergoing ileocecal resection. In a recent study, Schnitzler et al. showed that homozygosity of this variant together with active smoking is associated with a 100% risk for developing ileal stenosis requiring surgery [18]. In our analysis, previous immunosuppressive therapy, the homozygous NOD2 variant p.Leu1007fsX1008 and positive microscopic inflammation margins were not associated with increased risk for severe complications. This study identified male gender, the duration of CD, previous abdominal surgery, and the presence of fistulas as independent risk factors for the incidence of major postoperative complications following ileocolic resection in CD patients. One study by Giudici et al. also investigating NOD2 polymorphisms rs2066844, rs2066845, and rs2066847 in CD patients undergoing abdominal surgery in general could not identify homozygosity as risk factor for postoperative complications but a longer time period for disease onset in the p.Leu1007fsX1008 (rs2066847) cohort [19], whereas a study by Geramain et al. found the NOD2 SNP rs5743289 to be an independent risk factor for postoperative abdominal septic complications [20]. A third study by Kline et al. assessed the impact of NOD2 SNPs rs2076756, Arg702Trp (rs2066844), and p.Gly908Arg (rs2066845) [21]. In our cohort, rs2076756 was not assessed and the others were quite rare with only one homozygote Arg702Trp (rs2066844) and one p.Gly908Arg (rs2066845), respectively, but Kline et al. also did not find one of the NOD2 SNPs to be associated with postoperative complications after ileocecal resection [21]. In our follow-up data, we could not detect significant differences in the need for further surgical therapy or increased need for immunosuppressive or immune-modulatory therapy over time regarding the risk factors identified by others [18–20]: homozygous NOD2 variant, smoking, or fistulating disease.

There is an ongoing debate about the impact of immunosuppressive reagents on postoperative morbidity and the effect of preoperative steroids in particular. In this regard, the data remain rather inconsistent: in some studies, the use of steroids has been observed to increase the risk of postoperative septic complications [8, 12–14], whereas in other studies, including ours, this was not the case [6, 15–19]. This inconsistency may be explained by differences in the indications, duration, and dose of steroid treatment [14].

In addition to steroids, other immunomodulators have become standard therapy for CD [22]. In the present study, the influence of azathioprine and anti-TNF antibodies (infliximab, adalimumab) was investigated, and no increased risk was found. A recent systematic review by Law et al. [23] analyzed 63 studies regarding the effects of immunomodulators on postoperative complications after surgery for inflammatory bowel disease and found an increased risk for abdominal sepsis for anti-TNF medication and corticosteroids, whereas the risk was not increased for other immune-modulators and anti-integrins [23]. On the contrary, early initiation of immunomodulatory therapy postoperatively is recommended to reduce the risk of disease recurrence after surgery [24]. The initiation of anti-TNF therapy even in a period less than 2 weeks after surgery did not show an increased complication rate in a big retrospective cohort [25]. This seems to be important as a recent study has demonstrated an association between low anti-TNF drug levels and unfavorable postoperative outcomes in CD patients [26].

We know from several studies that poor nutritional status, such as low serum albumin or protein levels [27], low hemoglobin [28], or low BMI [29, 30], may put CD patients at risk for postoperative complications after abdominal surgery. As our cohort did not show clinical and laboratory signs of malnutrition, this can rather be excluded as a possible bias.

In our study, 22% of patients had a microscopically inflamed resection margin, although this was not a risk factor for complications in this analysis. Again, the data are rather heterogeneous: a recent observational study showed that CD patients with anastomotic leakage after elective ileocecal resection were more likely to have a positive resection margin [31], but our and other data [32] suggest that it is not necessary to achieve completely inflammation-free margins.

Homozygosity for the NOD2 frame shift mutation p.Leu1007fsX1008 has been described as a strong marker for a severe clinical phenotype of CD associated with more severe and penetrant disease behavior [1]. In children with CD, NOD2 mutation carrier status was associated with the need for surgery until 17 years of age [33]. Of the 114 patients with available NOD2 SNP status in our current study, 11 (9.6%) had the homozygous variant p.Leu1007fsX1008. This is twice as many as in a CD cohort from our colleagues in the Division of Gastroenterology [1], underscoring that surgery is more likely in this high-risk population of CD patients with this particular NOD2 frameshift mutation. However, this variant itself was not associated with higher morbidity in our study, indicating that the variant does not have an adverse effect on short-term postoperative outcome in these patients.

This study is subject to some limitations. Due to the retrospective design, the generalizability is limited as our data represent the experience from a single-center population and thereby reflect a small patient cohort. However, our study reports data from a genetically well-characterized CD population undergoing ileocolic resection with a long-term follow-up of median 123 month. Due to the data collecting period from 2001 to 2010, the patients did not receive further antibody treatment than anti-TNF. Therefore, this does not necessarily reflect todays situation, but this does not seem to be a strong bias since the overall complication rate of 40% is similar to those reported by others [8]. Nevertheless, redo-surgery was more frequent in our cohort with about 20% of cases. This can be explained by the high rate of fistulating disease in one-third of the cohort, which was significantly associated with the occurrence of postoperative complications. It is known that CD patients operated on in the acute stage are more likely to have an unfavorable postoperative course with about 40% increase of postoperative complications [8, 34]. The presence of preoperative fistulas as another surrogate parameter for severe and advanced disease progression has been identified by us and others [35–37] as a risk factor, as well as the duration of disease. It had been shown that patients undergoing early surgery had favorable disease course [38]. However, in the era of effective treatment options and interdisciplinary approaches, further studies will be required to figure out which patients will benefit from early surgical intervention and which will manage for a long time with immune-modulatory or immunosuppressive treatment. Therefore, the problem remains that surgeons often see patients too late when they are in a situation where only surgery can be recommended. In this regard, Iesalnieks et al. [6] and Alves et al. [39] reported that “recurrent clinical episodes” and “duration of symptoms leading to surgery” were associated with postoperative morbidity. Similarly, in our study, the time from initial diagnosis of CD to surgery was identified as an independent risk factor, with each year of CD duration increasing the risk by 11% per year. This confirms the importance of a multidisciplinary approach in which surgeons see patients early and repeatedly throughout the course of their disease.

In this study, fistulating disease was an important factor for performing open rather than laparoscopic resection. In our population, 59% of the open group and only 9% of the laparoscopic group had fistulating disease. Laparoscopic resection of the ileocolic is a commonly performed operation for CD that provides short-term benefits [39–41]. The laparoscopic approach for ileocecal CD has been increasingly used in recent years, especially in patients without fistulas or prior abdominal surgery. In our population, 65 patients (47%) underwent laparoscopic surgery, and 7 patients required conversion to open surgery. Overall, probably because of patient selection for the laparoscopic procedure (e.g., intestinal fistulas, previous abdominal surgery, age, and emergencies), patients with open surgery had a significantly higher morbidity rate (27% versus 12%). Of the 7 patients who required conversion, all had extensive abdominal adhesions that technically interfered with laparoscopy, and three had fistulas that had not been apparent preoperatively. No major complications occurred in this group. This is consistent with other publications in which no increased complication rate was observed in converted patients [6, 39]. Since adverse short- and long-term sequelae (especially recurrence) have been observed in patients with postoperative complications after abdominal surgery in CD, the identification of risk factors is of great importance, especially in the era of personalized medicine.

## Conclusion

Male gender, duration of CD, previous abdominal surgery, and fistulating disease but not NOD2 polymorphisms were identified as independent risk factors for the development of postoperative complications after ileocolic resection for CD. All of these risk factors should be considered in risk stratification to choose the best surgical approach (e.g., laparoscopic vs. open, stoma) for the individual patient.

## Data Availability

The data underlying this article cannot be published for ethical reasons to protect the privacy of the subjects included in the study. The data will be released in irreversibly anonymized form to the corresponding author upon justified request.

## Abbreviations

EBL: Estimated intraoperative blood loss
CD: Crohn’s disease
CT: Computer tomography
NOD2: Nucleotide oligomerization domain 2 (NOD2)
SNP: Single nucleotide polymorphism
TNF: Tumor necrosis factor

## References

[1] Schnitzler F, Friedrich M, Wolf C (2014). The NOD2 p.Leu1007fsX1008 mutation (rs2066847) is a stronger predictor of the clinical course of Crohn’s disease than the FOXO3A intron variant rs12212067. PLoS ONE.

[2] Torres J, Mehandru S, Colombel J-F, Peyrin-Biroulet L (2017). Crohn’s disease. The Lancet.

[3] Bernell O, Lapidus A, Hellers G (2000). Risk factors for surgery and postoperative recurrence in Crohn’s disease. Ann Surg.

[4] Caprilli R, Gassull MA, Escher JC (2006). European evidence based consensus on the diagnosis and management of Crohn’s disease: special situations. Gut.

[5] Vester-Andersen MK, Prosberg MV, Jess T (2014). Disease course and surgery rates in inflammatory bowel disease: a population-based, 7-year follow-up study in the era of immunomodulating therapy. Am J Gastroenterol.

[6] Iesalnieks I, Kilger A, Glaß H (2008). Intraabdominal septic complications following bowel resection for Crohn’s disease: detrimental influence on long-term outcome. Int J Colorectal Dis.

[7] Meima - van Praag EM, Buskens CJ, Hompes R, Bemelman WA,  (2021). Surgical management of Crohn’s disease: a state of the art review. Int J Colorectal Dis.

[8] European Society of Coloproctology collaborating group (2018). Risk factors for unfavourable postoperative outcome in patients with Crohn’s disease undergoing right hemicolectomy or ileocaecal resection. An international audit by ESCP and S-ECCO. Colorectal Dis.

[9] Büning C, Genschel J, Bühner S (2004). Mutations in the NOD2/CARD15 gene in Crohn’s disease are associated with ileocecal resection and are a risk factor for reoperation: NOD2/CARD15 Mutations are associated with ileocecal resection and reoperation in CD. Aliment Pharmacol Ther.

[10] Cuthbert AP, Fisher SA, Mirza MM (2002). The contribution of NOD2 gene mutations to the risk and site of disease in inflammatory bowel disease. Gastroenterology.

[11] Hugot J-P, Chamaillard M, Zouali H (2001). Association of NOD2 leucine-rich repeat variants with susceptibility to Crohn’s disease. Nature.

[12] Cario E (2005). Bacterial interactions with cells of the intestinal mucosa: toll-like receptors and NOD2. Gut.

[13] Saupe J, Berlin P, Reiner E et al (2021) Der Funktionsverlust des NOD2 Rezeptors verschlechtert die Anastomosenheilung nach Ileozökalresektion in der Maus. Z Gastroenterol 8. 10.1055/s-0041-1733542

[14] von Elm E, Altman DG, Egger M (2008). The strengthening the reporting of observational studies in epidemiology (STROBE) statement: guidelines for reporting observational studies. J Clin Epidemiol.

[15] Satsangi J (2006). The Montreal classification of inflammatory bowel disease: controversies, consensus, and implications. Gut.

[16] Dindo D, Demartines N, Clavien P-A (2004). Classification of surgical complications: a new proposal with evaluation in a cohort of 6336 patients and results of a survey. Ann Surg.

[17] Wirth U, Rogers S, Haubensak K (2018). Local antibiotic decontamination to prevent anastomotic leakage short-term outcome in rectal cancer surgery. Int J Colorectal Dis.

[18] Schnitzler F, Friedrich M, Angelberger M (2020). Development of a uniform, very aggressive disease phenotype in all homozygous carriers of the NOD2 mutation p.Leu1007fsX1008 with Crohn’s disease and active smoking status resulting in ileal stenosis requiring surgery. PLoS ONE.

[19] Giudici F, Cavalli T, Luceri C (2021). Long-term follow-up, association between CARD15/NOD2 polymorphisms, and clinical disease behavior in Crohn’s disease surgical patients. Mediators Inflamm.

[20] Germain A, Guéant R-M, Chamaillard M (2016). NOD2 gene variant is a risk factor for postoperative complications in patients with Crohn’s disease: a genetic association study. Surgery.

[21] Kline BP, Weaver T, Brinton DL (2020). Clinical and genetic factors associated with complications after Crohn’s ileocolectomy. Dis Colon Rectum.

[22] Terdiman JP, Gruss CB, Heidelbaugh JJ (2013). American Gastroenterological Association Institute Guideline on the use of thiopurines, methotrexate, and anti–TNF-α biologic drugs for the induction and maintenance of remission in inflammatory Crohn’s disease. Gastroenterology.

[23] Law CCY, Koh D, Bao Y (2020). Risk of postoperative infectious complications from medical therapies in inflammatory bowel disease: a systematic review and meta-analysis. Inflamm Bowel Dis.

[24] Bennett JL, Ha CY, Efron JE (2015). Optimizing perioperative Crohn’s disease management: role of coordinated medical and surgical care. WJG.

[25] Schad CA, Haac BE, Cross RK (2019). Early postoperative anti-TNF therapy does not increase complications following abdominal surgery in Crohn’s disease. Dig Dis Sci.

[26] Lau C, Dubinsky M, Melmed G (2015). The impact of preoperative serum anti-TNFα therapy levels on early postoperative outcomes in inflammatory bowel disease surgery. Ann Surg.

[27] Hennessey DB, Burke JP, Ni-Dhonochu T (2010). Preoperative hypoalbuminemia is an independent risk factor for the development of surgical site infection following gastrointestinal surgery: a multi-institutional study. Ann Surg.

[28] Tee MC, Shubert CR, Ubl DS (2015). Preoperative anemia is associated with increased use of hospital resources in patients undergoing elective hepatectomy. Surgery.

[29] Canedo J, Lee S-H, Pinto R (2011). Surgical resection in Crohn’s disease: is immunosuppressive medication associated with higher postoperative infection rates?: Surgical resection in Crohn’s disease. Colorectal Dis.

[30] Zhang M, Gao X, Yuanhan C (2015). Body mass index is a marker of nutrition preparation sufficiency before surgery for Crohn’s disease from the perspective of intra-abdominal septic complications: a retrospective cohort study erratum. Medicine.

[31] Poredska K, Kunovsky L, Marek F (2020). The influence of microscopic inflammation at resection margins on early postoperative endoscopic recurrence after ileocaecal resection for Crohn’s disease. Journal of Crohn’s and Colitis.

[32] Pennington L, Hamilton S, Bayless T, Cameron J (1980). Surgical management of Crohn’s disease. Ann Surg.

[33] Lacher M, Helmbrecht J, Schroepf S (2010). NOD2 mutations predict the risk for surgery in pediatric-onset Crohn’s disease. J Pediatr Surg.

[34] Udholm LS, Rasmussen SL, Madsbøll TK (2021). A systemic review and metaanalysis of postoperative outcomes in urgent and elective bowel resection in patients with Crohn’s disease. Int J Colorectal Dis.

[35] Post S, Betzler M, von Ditfurth B (1991). Risks of intestinal anastomoses in Crohn’s disease. Ann Surg.

[36] Yamamoto T, Allan RN, Keighley MRB (2000). Risk factors for intra-abdominal sepsis after surgery in Crohn’s disease. Dis Colon Rectum.

[37] Guo K, Ren J, Li G (2017). Risk factors of surgical site infections in patients with Crohn’s disease complicated with gastrointestinal fistula. Int J Colorectal Dis.

[38] Lee JM, Lee K-M, Kim JS (2018). Postoperative course of Crohn disease according to timing of bowel resection: results from the CONNECT Study. Medicine.

[39] Alves A, Panis Y, Bouhnik Y (2005). Factors that predict conversion in 69 consecutive patients undergoing laparoscopic ileocecal resection for Crohn’s disease: a prospective study. Dis Colon Rectum.

[40] Polle SW, Wind J, Ubbink DT (2006). Short-term outcomes after laparoscopic ileocolic resection for Crohn’s disease. Dig Surg.

[41] Fichera A, Peng SL, Elisseou NM (2007). Laparoscopy or conventional open surgery for patients with ileocolonic Crohn’s disease? A prospective study. Surgery.

